# Effects of combined deferiprone with deferoxamine on right ventricular function in thalassaemia major

**DOI:** 10.1186/1532-429X-14-8

**Published:** 2012-01-25

**Authors:** Francisco Alpendurada, Gill C Smith, John-Paul Carpenter, Sunil V Nair, Mark A Tanner, Winston Banya, Carlo Dessi, Renzo Galanello, John Malcolm Walker, Dudley J Pennell

**Affiliations:** 1 Royal Brompton & Harefield NHS Foundation Trust, London, UK; 2 Imperial College, London, UK; 3 University College Hospital, London, UK; 4 Ospedale Regionale per le Microcitemie, Cagliari, Italy

**Keywords:** thalassaemia major, deferiprone, deferoxamine, right ventricular function

## Abstract

**Background:**

Combination therapy with deferoxamine and oral deferiprone is superior to deferoxamine alone in removing cardiac iron and improving left ventricular ejection fraction (LVEF). The right ventricle (RV) is also affected by the toxic effects of iron and may cause additional cardiovascular perturbation. We assessed the effects of combination therapy on the RV in thalassaemia major (TM) using cardiovascular magnetic resonance (CMR).

**Methods:**

We retrieved imaging data from 2 treatment trials and re-analyzed the data for the RV responses: Trial 1 was a randomized controlled trial (RCT) of 65 TM patients with mild-moderate cardiac siderosis receiving combination therapy or deferoxamine with placebo; Trial 2 was an open label longitudinal trial assessing combination therapy in 15 TM patients with severe iron loading.

**Results:**

In the RCT, combination therapy with deferoxamine and deferiprone was superior to deferoxamine alone for improving RVEF (3.6 vs 0.7%, p = 0.02). The increase in RVEF was greater with lower baseline T2* 8-12 ms (4.7 vs 0.5%, p = 0.01) than with T2* 12-20 ms (2.2 vs 0.8%, p = 0.47). In patients with severe cardiac siderosis, substantial improvement in RVEF was seen with open-label combination therapy (10.5% ± 5.6%, p < 0.01).

**Conclusions:**

In the RCT of mild to moderate cardiac iron loading, combination treatment improved RV function significantly more than deferoxamine alone. Combination treatment also improved RV function in severe cardiac siderosis. Therefore adding deferiprone to deferoxamine has beneficial effects on both RV and LV function in TM patients with cardiac siderosis.

## Background

In transfusion-dependent thalassaemia major (TM) patients, iron chelation therapy is mandatory to prevent or reverse iron accumulation caused by excess intake from transfusional iron and the increased gastrointestinal absorption. Deferoxamine was the first clinically available iron chelating agent, introduced over 40 years ago, and life expectancy in TM increased dramatically with its use. [1,2] However, its beneficial effects are tempered by the cumbersome treatment regimes required, which may be a contributor to the frequently observed long term complications of heart failure and cardiac death. [3]

Deferiprone is an orally active chelator with a lower molecular weight that is uncharged at physiological pH, and which is both hydrophilic and lipophilic enabling it to readily penetrate myocardial cells. It has been shown to be superior to deferoxamine in removing iron from the myocardium, and is associated with improved cardiac outcomes. [4-9] Due to differences in their access to body iron pools, the use of a combination of the two chelators seems to have a synergistic effect on removal of excess iron. [9,10] A recent randomised controlled trial comparing combination therapy with subcutaneous deferoxamine and oral deferiprone against deferoxamine monotherapy showed combination treatment to be superior in removing cardiac iron and improving left ventricular ejection fraction (LVEF). [11] The beneficial effects of combination therapy on LVEF have also been confirmed in patients with TM and severe iron loading. [12]

However, despite this success for LV function, the importance of combination therapy on right ventricular (RV) function has not been reported, even though the RV can be affected by the toxic effects of myocardial iron. [13,14] Cardiovascular magnetic resonance (CMR) provides highly reliable and reproducible measurements of RV volumes and function as well as myocardial iron using the T2* method. [15,16] We therefore compared the effects of combination treatment (deferoxamine and deferiprone) with deferoxamine monotherapy on RV function in TM patients with cardiac iron overload.

## Methods

### Study population

**Figure 1:**
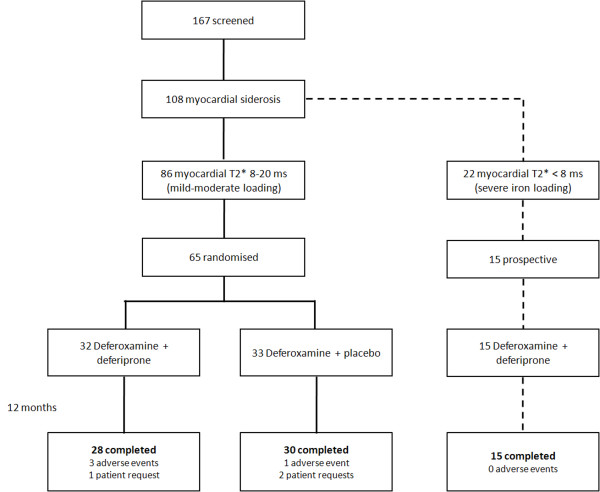
**Study flow-chart**.

In order to examine the effects of combination treatment on the RV, we reanalyzed imaging data from 2 previously reported trials. The first was a randomized, double-blind, placebo controlled trial (RCT) comparing combined therapy of deferoxamine with deferiprone against deferoxamine with placebo in mild-moderate myocardial siderosis. [11] The second trial was a longitudinal open-label study of combination treatment (no comparison arm) in patients with severe cardiac siderosis and impaired LV function. [12] Both trials were run simultaneously in Cagliari Italy (Figure 1). The study protocol was approved by ethics committees in London and Cagliari. Patient information and consent forms were in Italian and all patients gave written informed consent. [11,12] Brief details of the trials are given below.

In the RCT, 167 adult TM patients (75 males, mean age 30 ± 5.3 years) were screened for quantification of myocardial iron loading using myocardial T2*. Inclusion criteria for patient screening were: diagnosis of TM currently maintained on subcutaneous deferoxamine monotherapy; age > 18 years; and maintaining pre-transfusion haemoglobin > 9 g/dL. Exclusion criteria were: patients who had received deferiprone for a total of > 6 months over the last 5 years; patients with previous reaction to deferiprone; neutropenia (absolute neutrophil count < 1.5 × 10^9^/L) at screening; thrombocytopenia ( < 50 ×10^9^/L) at screening; liver enzymes > 3 times upper limit of normal; any condition making CMR impossible or inadvisable. Of the 167 patients screened, 108 had significant myocardial siderosis (T2* < 20 ms), of whom 22 (13%) had severe myocardial loading (T2* < 8 ms). Patients with mild to moderate cardiac iron loading who satisfied the trial entry criteria (myocardial T2* 8-20 ms, n = 86) were invited for further detailed assessment by CMR. Of these, 65 were subsequently randomized to receive either deferoxamine plus deferiprone (combined group; n = 32) or deferoxamine plus placebo (deferoxamine group; n = 33), and were followed-up for 12 months.

Patients with severe cardiac siderosis (T2* < 8 ms) were excluded from the RCT and it was at the treating clinician's discretion to determine best clinical practice for chelation therapy. Of the 22 patients with severe myocardial siderosis, 15 (9 females, 28.9 ± 4.8 years) received open-label combination therapy according to locally developed protocols, and were followed prospectively over one year. These patients were used in a secondary comparative analysis against patients from the randomised trial who were on combination therapy.

### Cardiovascular magnetic resonance

A mobile 1.5 Tesla CMR scanner (Sonata, Siemens Medical Systems, Erlangen, Germany) was transported to Cagliari for this research. Myocardial and hepatic T2* were assessed using the bright-blood single breath-hold multi-echo technique as previously described. [17] T2* analysis was performed using Thalassaemia-Tools (a plug-in of CMRtools, Cardiovascular Imaging Solutions, London, UK) with curve truncation to account for background noise. [18] Right ventricular volumes and ejection fraction were determined at baseline and at 12 months of treatment with steady state free precession cines using contiguous short-axis slices from base to apex. [15] CMRtools was used for RV volume analysis. These measurements were performed by observers blinded to the patient's clinical details and chelation regime.

### Echocardiography

Doppler echocardiography studies were performed at baseline and at 12 months to look for pulmonary hypertension. Pulmonary artery systolic pressures (PAP) were determined by peak velocity of the tricuspid regurgitation jet plus estimation of right atrial pressures using standard methodology. Pulmonary hypertension was defined as PAP > 40 mmHg.

### Biochemistry

Laboratory measures included weekly full blood count (due to the risk of agranulocytosis with deferiprone), serum ferritin (Abbott AXSYM System), B-type natriuretic peptide (BNP-Biosite Diagnostics Inc, San Diego, California), and liver function tests (alanine aminotransferase - ALT).

### Statistical Analysis

Categorical data are presented as frequency and percentage (%). Continuous variables are presented as mean ± standard deviation (SD), except for BNP, which is displayed in median and interquartile range; and for T2* and ferritin, which use the geometric mean (anti-log of the mean of the log data) ± coefficient of variation (CV). Baseline characteristics of both treatment groups were compared using an unpaired two-tailed t-test for continuous variables (except for BNP, which was compared by a non-parametric test) and a chi-squared test for categorical variables. Analysis of variance (ANOVA) was used to compare changes in T2* and RVEF over 12 months with treatment and baseline measures entered as covariates. Changes in RVEF over 12 months within individual groups were compared with a paired t-test. Correlations of myocardial T2* with ventricular function were performed using the Spearman's rank test. Subgroup analysis was performed according to severity of myocardial iron loading with cut-offs of 8 ms and 12 ms used to define patients with mild (T2* 12-20 ms), moderate (T2* 8-12 ms) and severe (T2* < 8 ms) iron loading. Intraobserver and interobserver variability was assessed using the method of Bland and Altman. [19] The coefficient of variability was calculated as the SD of the differences between two sets of measurements divided by the mean value of the parameter under consideration. Statistical significance was set at p < 0.05. All statistical analysis was performed using Stata 10.1 software (StataCorp, Texas, USA).

## Results

### RCT in mild to moderate cardiac siderosis

The baseline findings and the results of the RCT comparing combination treatment against deferoxamine alone for changes in myocardial T2* and LV EF have been previously published,[11] and are briefly summarized here (table 1). The patients randomized to combination therapy or deferoxamine alone were evenly matched at baseline. The prescribed dose of deferiprone in the combination arm was 75 mg/kg/day. The average dose of deferoxamine in the deferoxamine alone arm (40.5 mg/kg/day for 5 days/week) was comparable to the combination arm (40.6 mg/kg/day for 5 days/week, p = 1.0). Four patients in the combination arm withdrew from the study (3 due to adverse events), and 3 patients in the deferoxamine arm withdrew (1 due to an adverse event). Thus, 28 patients in the combination arm and 30 patients in the deferoxamine alone arm completed the study. Over 12 months, the combination treatment group showed superior improvement in myocardial T2* compared with the deferoxamine group (ratio of change in geometric means 1.50 vs. 1.24, p = 0.02).

**Table 1:** Baseline characteristics of the randomized controlled trial population, according to treatment arm.

	Combined	Deferoxamine	p-value
Number of patients	32	33	-

Age (years)	28.8 ± 4.2	28.7 ± 5.3	0.9

Gender (male)	14 (44%)	13 (39%)	0.5

Body surface area	1.53 ± 0.15	1.56 ± 0.16	0.5

Heart rate	78 ± 10	81 ± 16	0.3

Deferoxamine dose (mg/kg/day)	40.6 ± 13.2 (5 days/week)	40.5 ± 14.0 5 days/week)	1.0

**CMR measures**			

Myocardial T2*	11.7 (0.08)	12.4 (0.11)	0.3

Liver T2*	4.9 (0.52)	4.2 (0.62)	0.5

RVEDV (mL)	129.5 ± 30.8	131.8 ± 34.4	0.8

RVESV (ml)	52.5 ± 17.5	52.3 ± 18.5	1.0

RVEF (%)	60.2 ± 7.2	61.0 ± 7.1	0.7

**Echo measures**			

PAP (mmHg)	22.3 ± 5.0	20.9 ± 5.4	0.4

**Blood measures**			

Transfusional red blood cell input (mL/kg/year)	133.4 ± 34.9	130.2 ± 38.6	0.7

Haemoglobin (g/L)	106 ± 9.6	102 ± 9.5	0.1

Hepatitis C positive	23 (72%)	26 (79%)	0.4

Serum ferritin (μg/L)	1574 (11)	1379 (10)	0.5

BNP (pmol/L)	13.6 (5.6, 30.1)	15.2 (7.3, 26.2)	0.7

Creatinine (mg/dL)	0.77 ± 0.21	0.74 ± 0.23	0.6

**Cardiac medication**			

Any	5 (16%)	8 (24%)	0.4

Digoxin	3 (9%)	4 (12%)	0.7

ACEi/ARB	5 (16%)	8 (24%)	0.4

Diuretics	3 (9%)	5 (15%)	0.5

Values are presented as n (%), mean ± SD, geometric mean (coefficient of variation), or as median (25^th ^- 75^th ^percentile). RV = right ventricular; EDV = end-diastolic volume; ESV = end-systolic volume; PAP = pulmonary artery systolic pressure; BNP = B-type natriuretic peptide. ACEi = angiotensin-converting enzyme inhibitors; ARB = angiotensin II receptor blockers.

**Figure 2:**
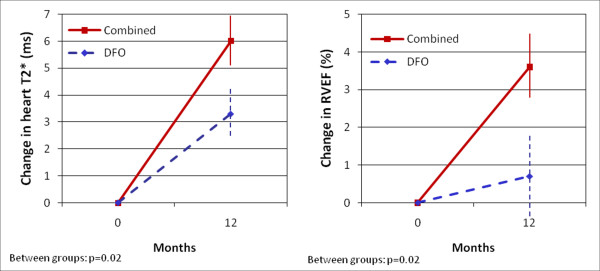
Change in myocardial T2* (left panel) and in RVEF (right panel) over 12 months according to treatment arm. Vertical lines represent standard error.

In the combination group RVEF increased from 60.2 ± 7.2% at baseline to 63.8 ± 5.9% at 12 months (p < 0.01), whereas in the deferoxamine group RVEF did not change significantly (61.0 ± 7.1% at baseline vs. 61.7 ± 6.4% at 12 months, p = 0.49). There was a significant difference in the RVEF response between groups favouring combination therapy (3.6 vs. 0.7%, p = 0.02; Figure 2). The improvement in RVEF in the combined group was mainly driven by a decrease in RV end-systolic volumes (52.5 ± 17.5 ml to 46.4 ± 14.9 ml, p < 0.01) rather than a change in RV end-diastolic volumes (129.5 ± 30.8 ml to 126.1 ± 29.8 ml, p = 0.20). There was no significant change in PAP between the combination vs. the deferoxamine arm from baseline to one year (-1.8 mmHg vs. +0.3 mmHg, p = 0.19).

**Figure 3:**
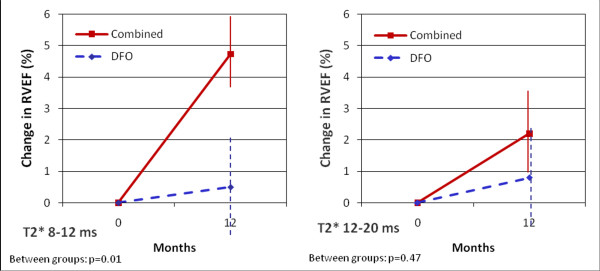
Change in RVEF over 12 months according to treatment arm and myocardial T2* at baseline (T2* 8-12 ms on left panel, T2* 12-20 ms on right panel). Vertical lines represent standard error.

The median myocardial T2* in the RCT at baseline was 12.0 ms. This value was used as a cut-off to define patients with mild (myocardial T2* 12-20 ms) and moderate iron loading (myocardial T2* 8-12 ms), and this was in accord with the cut-offs used in the original trials. Both subgroups were then analysed according to the chelation regime. Comparing the individual treatment arms, we observed a significant improvement in RVEF in patients with T2* between 8 and 12 ms on combination therapy (58.5 ± 6.9% at baseline vs 63.3 ± 6.0% at 12 months, p < 0.01), and borderline significant improvement in patients with T2* between 12 and 20 ms (62.1 ± 7.3% at baseline vs. 64.3 ± 6.0% at 12 months, p = 0.08). Conversely, patients on deferoxamine alone had no significant improvement in RVEF, whether the baseline T2* was 8-12 ms (59.3 ± 6.8% at baseline vs. 59.8 ± 6.4% at 12 months, p = 0.70) or 12-20 ms (62.5 ± 7.2% at baseline vs. 63.3 ± 6.1% at 12 months, p = 0.58). Comparison of the between groups effects showed that combination therapy was superior to deferoxamine alone in improving RVEF in patients with baseline T2* below 12 ms (4.7% vs. 0.5%, p = 0.01) but not in those with T2* above 12 ms (2.2% vs. 0.8%, p = 0.47; Figure 3).

Intraobserver and interobserver variability of the right ventricular volumes and ejection fraction was derived from the first 20 subjects participating in the study (table 2). The coefficient of variability for the right ventricular measurements was small, in keeping with previous publications on the same area. [20,21]

**Table 2:** Intraobserver and interobserver reproducibility for the right ventricle.

	Intraobserver		Interobserver	
	
	Mean difference (± SD)	CoV (%)	Mean difference (± SD)	CoV (%)
RV end-diastolic volume (mL)	0.6 ± 5.0	3.9	6.7 ± 8.9	7.1

RV end-systolic volume (mL)	-0.1 ± 3.6	6.7	2.0 ± 4.7	8.9

RV ejection fraction (%)	0.5 ± 1.9	3.2	1.0 ± 3.0	5.2

CoV = coefficient of variability; RV = right ventricle.

### Longitudinal open-label study in severe cardiac siderosis

The baseline findings and the results of the longitudinal study in the 15 patients with severe iron loading (myocardial T2* < 8 ms) evaluating combination treatment for changes in myocardial T2* and LVEF have been previously published,[12] and are briefly summarized in table 3. Two patients were in clinical heart failure, and both had BNP levels > 100 pmol/l. The mean prescribed doses of deferoxamine and deferiprone at baseline were 38.0 mg/kg for 5.3 days/week (equivalent to 40.7 mg/kg for 5 days/week) and 73.9 mg/kg/day, respectively. During the trial, doses were reduced to 20.3 mg/kg for 4.5 days/week and 65.7 mg/kg/day respectively, primarily due to reductions in ferritin. [11] All 15 patients received unblinded combination therapy with deferoxamine and deferiprone throughout the study period. Over 12 months, there was a significant improvement in myocardial T2* (ratio of change in geometric means 1.31, p < 0.01).

**Table 3:** Baseline characteristics of the randomized controlled trial population (mild and moderate myocardial iron loading) vs. open-label combination therapy population (severe myocardial iron loading). Values and abbreviations presented as in table 1.

	Mild-moderate siderosis	Severe siderosis	p-value
	**RCT**	**Longitudinal trial**	

Number	65	15	-

Age (years)	28.8 ± 4.7	27.8 ± 4.8	0.5

Gender (male)	27 (41.5%)	6 (40.0%)	0.9

Body surface area	1.55 ± 0.15	1.56 ± 0.13	0.8

Heart rate	79 ± 13	97 ± 18	< 0.01

Deferoxamine (mg/kg/day)	40.6 ± 13.5 (5 days/week)	40.7 ± 12.0 (5 days/week)	0.9

**CMR measures**			

Myocardial T2*	12.0 (0.13)	6.0 (0.09)	N/A

Liver T2*	4.5 (0.57)	2.9 (0.67)	0.06

RVEDV (mL)	130.7 ± 32.4	146.7 ± 35.1	0.2

RVESV (mL)	52.4 ± 17.8	76.5 ± 27.6	< 0.01

RVEF (%)	60.6 ± 7.1	49.0 ± 9.4	< 0.01

**Echo measures**			

PAP (mmHg)	21.6 ± 5.2	22.0 ± 5.9	0.8

**Blood measures**			

Hepatitis C positive	49 (75%)	11 (73%)	0.9

Serum ferritin (μg/L)	1472 (0.11)	2057 (0.08)	0.1

BNP (pmol/L)	15.0 (7.1, 28.8)	26.0 (15.6, 40.5)	0.01

**Cardiac medication**			

Any	13 (20%)	7 (47%)	0.03

Digoxin	7 (11%)	4 (27%)	0.1

ACEi/ARB	13 (20%)	7 (47%)	0.03

Diuretics	8 (12%)	4 (27%)	0.2

In 12 of the 15 patients (80%), the baseline RVEF was low compared to a reference TM population with a normal T2*. [22] The baseline RVESV was significantly raised and there was a trend for a higher RVEDV, resulting in a significantly reduced mean RVEF (49.0 ± 9.4%) when compared to the population with less severe iron loading (table 2). The open-label group was more frequently medicated for heart failure, particularly angiotensin-converting enzyme inhibitors (ACEi)/angiotensin II receptor blockers (ARB). No patients in the RCT or open-label cohorts were on beta-blockers. Pulmonary artery systolic pressures were similar in both groups (22.0 ± 5.9 mmHg vs 21.6 ± 5.2 mmHg, p = 0.82). Over 12 months, there was a significant improvement in RVEF (10.5 ± 5.6%, p < 0.01), with no significant change in PAP (22.0 mmHg vs 24.4 mmHg at 12 months, p = 0.16). No new cardiac medications were commenced during the study (except for the 2 patients presenting with heart failure, who were asymptomatic by the end of the study). Despite a positive trend, neither cardiac medication as a whole (increase RVEF 12 ± 6% vs 9 ± 5%, p = 0.2), nor individual medications (digoxin: 14 ± 3% vs 10 ± 6%, p = 0.2; ACEi/ARB: 12 ± 6% vs 9 ± 5%, p = 0.2; diuretics: 13 ± 5% vs 10 ± 6%, p = 0.4) were significantly associated with a higher increase in RVEF compared with those without cardiac medication.

**Figure 4:**
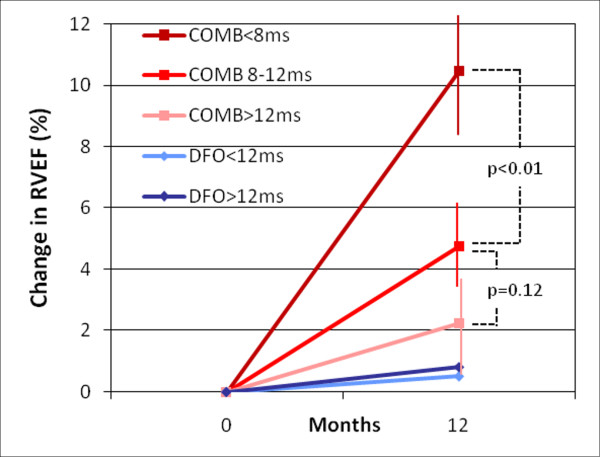
Breakdown of improvement in RVEF (%) for different groups according to chelation therapy and myocardial T2* baseline. Vertical lines represent standard error.

When grouping the patients on combination therapy (the 32 patients from the combination arm in the RCT plus the 15 unblinded patients on open-label combined therapy), we found an inverse relation between myocardial T2* at baseline and improvement in RVEF over one year (Figure 4). Patients with severe iron loading had a greater improvement in RVEF than patients with moderate iron loading (10.5% vs. 4.7%, p < 0.01).

### Entire study cohort

**Figure 5:**
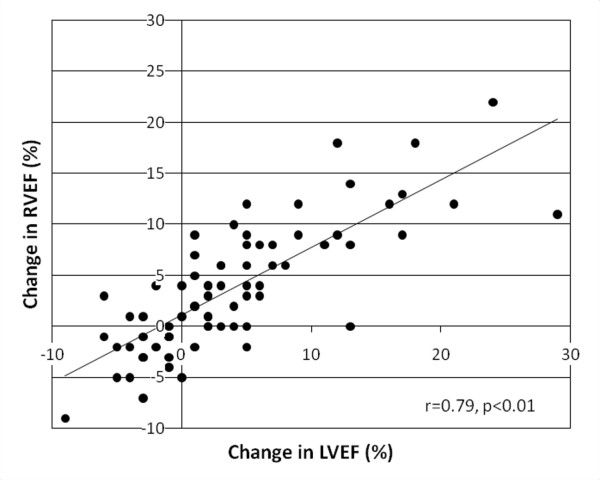
**Correlation of change in RVEF with change in LVEF over 12 months**.

In the cohort of 80 patients with myocardial iron loading, a significant correlation was observed between baseline myocardial T2* and baseline RVEF (r = 0.46, p < 0.01) and baseline LVEF (r = 0.50, p < 0.01). Accordingly, there was a strong correlation between RVEF and LVEF at baseline (r = 0.82, p < 0.01). The improvement in RVEF during the study period also correlated with the improvement in LVEF (r = 0.79, p < 0.01; Figure 5). We also analyzed the change in RVEF with the change in cardiac iron as derived from recent human cardiac iron calibration data from Carpenter et al. [23] There was a significant inverse correlation between the improvement in myocardial iron concentration and the improvement in RVEF (r = -0.42, p < 0.01).

## Discussion

With the development of the T2* technique, CMR has provided new insights into iron-overload cardiomyopathy, as the myocardial iron concentration and its toxic effect on ventricular function can be assessed at the same time with the same high-fidelity technique. In the first T2* publication by Anderson et al, normal T2* levels were associated with normal LVEF, but when T2* fell below 20 ms, there was a progressive fall in LVEF, showing that increasing iron loading is associated with worsening of LV function. [16] Similar observations have recently been made for the RV,[14] suggesting RV dysfunction may be a contributor to heart failure and cardiac mortality in TM patients, as has been found in other cardiac conditions. [24-28] However, to date, there has been little published data on the response of the right ventricle to chelation therapy, and no data at all on the most appropriate chelation regime in the presence of right ventricular dysfunction.

Chelation with deferoxamine has been one of the cornerstones for the treatment of TM. It has been extensively studied over the past decades and has shown to decrease the total body iron burden, prevent complications of iron overload and improve survival in TM. [1,2,29] However, long-term deferoxamine monotherapy has been hampered by poor compliance and failure of long term prevention of myocardial iron deposition, heart failure and cardiac deaths. [4,5] Deferiprone is a more recent iron chelator which has a lower molecular weight, is more lipophilic, is uncharged at physiologic pH and consequently appears better able to penetrate cells and organelles than deferoxamine. This may in part explain why deferiprone is superior to deferoxamine for removing iron from the heart. [6] The combined use of these two chelating agents which exploits the relative merits of each drug, has been supported in animal models, and has become an attractive therapeutic option in severe cardiac iron loading or when negative iron balance has not been achieved by other methods. [11,30,31] Observational, prospective and randomised controlled studies have demonstrated the efficacy of combined therapy in removing iron from the liver and heart, improving endothelial function and left ventricular function,[11,12] as well as endocrine function. [32] Our current study compared the effects of combination therapy on RV function in TM patients with myocardial iron loading. Our findings from the RCT show combination therapy to be superior to subcutaneous deferoxamine alone in improving RV function in patients with mild and moderate iron loading. In addition, our data from the longitudinal open-label study show that RV dysfunction is reversible even in patients with severe iron overload. It is of interest that the recovery in RV function was greatest in patients with a more severe degree of myocardial siderosis and RV dysfunction.

It is well recognized that RV performance depends not only on intrinsic contractility but also on RV afterload, which is the resistance that the RV has to overcome during ejection. Increased pulmonary artery pressures reflecting increased pulmonary vascular resistance may thus impair RV function. [33] However, none of the participants in this study had pulmonary hypertension, in line with previous studies suggesting a low prevalence of pulmonary hypertension in TM. [34,35] Whilst an improvement in myocardial T2* was associated with an improvement in RVEF, no significant changes in pulmonary artery pressures were observed throughout the study. Therefore, the improvement in RV function seen in this population was independent of pulmonary artery pressure thus eliminating a potential confounding factor for the evaluation of RV function.

Our data are unusual, because reversible RV dysfunction in the setting of a cardiomyopathy is a rarely documented phenomenon. The magnitude of recovery in RV function parallels the recovery in LV function, and this correlates with the response to chelation therapy. This is in keeping with the findings of other studies in non-ischaemic cardiomyopathies, where it has been observed that LV and RV function is usually affected in a similar way and to a similar extent. [24,36] The close association seen between RVEF and LVEF and the increase of these parameters over time following successful myocardial iron chelation supports the concept that intrinsic RV myocardial contractility is predominantly affected by intracellular iron and plays a key role in RV performance in this toxic-induced cardiomyopathy model. [14] Finally, as right ventricular ejection fraction has incremental prognostic value which is additional to LVEF,[24-27] it is reasonable to suggest that the improvement in RVEF seen in this study with the combined use of deferiprone and deferoxamine may contribute to improved outcomes.

### Limitations

This is a retrospective analysis of 2 trials designed to assess the change on myocardial T2* with iron chelation regimes in which RV parameters were not planned as primary end-points. Nevertheless, all data was prospectively collected and the current RV analysis was blinded to the patients' details and chelation regimes.

## Conclusions

In the RCT of mild to moderate cardiac iron loading, combination treatment improved RV function significantly more than deferoxamine alone. Combination treatment also improved RV function in severe cardiac siderosis. Therefore adding deferiprone to deferoxamine has beneficial effects on both RV and LV function in TM patients with cardiac siderosis.

## Competing interests

FA has received speaker's honoraria from Novartis. GCS is a consultant to Novartis. DJP is a consultant to Novartis and ApoPharma, and a director of Cardiovascular Imaging Solutions. DJP has received research support and speaker's honoraria from Siemens, Novartis, and ApoPharma. JPC has received speaker's honoraria from Swedish Orphan and ApoPharma. SN has received financial assistance from both Novartis and Swedish Orphan for attendance at conferences.

## Authors' contributions

FA and GCS participated in the design of the study, data collection, and drafted the manuscript. JPC, SVN and MAT participated in the data collection. WB performed the statistical analysis. CD, RG, JMW helped to draft the manuscript. DJP conceived of the study, participated in the design of the study and helped to draft the manuscript. All authors read and approved the final manuscript.
